# *Poncirus trifoliata* Aqueous Extract Protects Cardiomyocytes against Doxorubicin-Induced Toxicity through Upregulation of NAD(P)H Dehydrogenase Quinone Acceptor Oxidoreductase 1

**DOI:** 10.3390/molecules28248090

**Published:** 2023-12-14

**Authors:** Min-Sun Kim, Hyo-Kyoung Choi, Soo-Hyun Park, Jae-In Lee, Jangho Lee

**Affiliations:** Korea Food Research Institute, Wanju 55365, Republic of Korea; mskim@kfri.re.kr (M.-S.K.); chkyoung@kfri.re.kr (H.-K.C.); shpark0204@kfri.re.kr (S.-H.P.); jaeinlee@kfri.re.kr (J.-I.L.)

**Keywords:** doxorubicin-induced cardiotoxicity, oxidative stress, apoptosis, *Poncirus trifoliza*

## Abstract

Doxorubicin (DOX), an anthracycline-based chemotherapeutic agent, is widely used to treat various types of cancer; however, prolonged treatment induces cardiomyotoxicity. Although studies have been performed to overcome DOX-induced cardiotoxicity (DICT), no effective method is currently available. This study investigated the effects and potential mechanisms of *Poncirus trifoliata* aqueous extract (PTA) in DICT. Changes in cell survival were assessed in H9c2 rat cardiomyocytes and MDA-MB-231 human breast cancer cells. The C57BL/6 mice were treated with DOX to induce DICT in vivo, and alterations in electrophysiological characteristics, serum biomarkers, and histological features were examined. The PTA treatment inhibited DOX-induced decrease in H9c2 cell viability but did not affect the MDA-MB-231 cell viability. Additionally, the PTA restored the abnormal heart rate, R-R interval, QT interval, and ST segment and inhibited the decrease in serum cardiac and hepatic toxicity indicators in the DICT model. Moreover, the PTA administration protected against myocardial fibrosis and apoptosis in the heart tissue of mice with DICT. PTA treatment restored DOX-induced decrease in the expression of NAD(P)H dehydrogenase quinone acceptor oxidoreductase 1 in a PTA concentration-dependent manner. In conclusion, the PTA inhibitory effect on DICT is attributable to its antioxidant properties, suggesting the potential of PTA as a phytotherapeutic agent for DICT.

## 1. Introduction

Recent projections suggest that the global incidence of cancer will reach 26 million by 2040, with 15 million individuals requiring chemotherapy; the most common types of cancer requiring chemotherapy are lung (16.4%), breast (12.7%), and colorectal (11.1%) cancers [1]. Doxorubicin (DOX), approved by the USA Food and Drug Administration approval in 1974, is widely used as a first-line therapy, either as a stand-alone drug or in combination with other anticancer drugs, for the treatment of various cancers, including breast [2], ovarian [3], gastric [4], and lung [5] cancers.

Despite its significant side effects, DOX remains in use because of its effectiveness in cancer treatment. DOX-induced cardiotoxicity (DICT) is an undesired and irreversible severe side effect that leads to left ventricular dysfunction and congestive heart failure [6], increasing the risk of mortality among patients with cancer. Multiple mechanisms have been suggested for DICT, with free radical generation, oxidative stress, and apoptosis being the most proposed pathways [7]. Particularly, oxidative stress plays a significant role in triggering DICT, as it results in excessive generation of reactive oxygen species (ROS) and consequently in cellular alterations and damage [8]. Moreover, the production of superoxide anions (O_2_^−^) occurs during the redox cycling of DOX or as a result of oxido-reduction reactions involving the anthracycline-iron complex. Furthermore, redox cycling of the DOX quinone ring can be facilitated by various NAD(P)H dehydrogenases and oxidases [9]. 

NAD(P)H dehydrogenase quinone acceptor oxidoreductase 1 (NQO1), also known as DT-diaphorase, is a versatile protein with multiple protective functions in addition to its catalytic role [10]. NQO1 is a widely distributed FAD-dependent flavoprotein that plays a crucial role in catalyzing the reduction of various substances, including quinones, quinone imines, nitroaromatics, and azo dyes [11]. The intracellular biological functions of NQO1 are highly diverse and include superoxide scavenging, xenobiotic metabolization, and inflammatory signal modulation [12,13]. One of the primary protective roles of NQO1 is its direct anti-oxidant activity via the two-electron reduction of various quinones into their corresponding hydroquinones, using either NADPH or NADH as the source of hydride ions [14]. NQO1 is highly expressed in mammalian organs, with the most significant amounts found in the liver, kidney, and cardiovascular systems [15]. Recent research has revealed promising roles for NQO1 in protecting against cardiovascular damage [16,17]. Similar to other antioxidant enzymes, such as superoxide dismutase 1 (SOD1), glutathione peroxidase (GPX) 3, GPX4, and peroxidedoxin 1, the expression of NQO1 decreases heart failure [18]. The possible role of NQO1 in chemoprotection has been extensively examined in numerous reviews [19,20], and extensive studies have been performed on its structure and mechanisms [14,21].

*Poncirus trifoliata* (L.) Raf is a member of the family Rutaceae and belongs to the genus *Poncirus*, which is closely related to but distinct from the Citrus genus. *P. trifoliata* has been used in traditional medicine for centuries and is known for its anti-allergy, antimicrobial, antifungal, antilipidemic, and anti-inflammatory activities [22]. Additionally, *P. trifoliata* extract exhibits cytotoxic effects against cancer cells; moreover, *P. trifoliata* suppresses the proliferation, migration, and invasion of the human hepatocarcinoma cell lines Hep3B and Huh7 [22]. Notably, *P. trifoliata* inhibited the growth of the human colon cancer cell line HT-29 [23] and breast cancer cell lines MDA-MB-231 and BT-483 [24], and promote programmed cell death in the gastric cancer cell line AGS [25] and human leukemia cell line HL-60 [26]. However, the effects and molecular mechanisms of *P*. *triforliata* on DICT are yet to be elucidated. 

Therefore, this study aimed to elucidate the cardioprotective and cardiac function-preserving effects of *Poncirus trifoliata* aqueous extract (PTA) in cardiomyocytes and a mouse model of DICT. Additionally, a PCR microarray was performed to identify potential target genes involved in the protective effects of PTA.

## 2. Results

### 2.1. PTA Inhibits DOX-Induced Cytotoxicity in H9c2 Cells 

**Figure 1:**
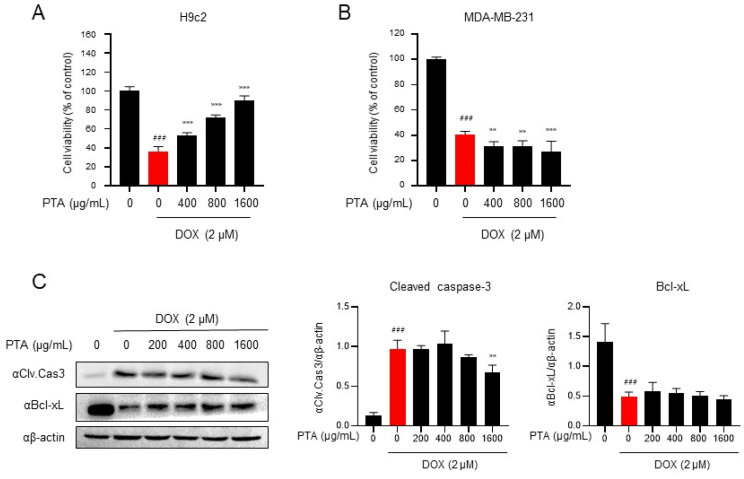
*Poncirus trifoliata* aqueous extract (PTA) decreases doxorubicin (DOX)-induced cellular toxicity in rat cardiomyocytes (H9c2 cells), without interference with the anticancer effects of DOX. (**A**) PTA protects H9c2 cell viability against DOX-induced toxicity. H9c2 cells were pretreated with specific concentrations of PTA (0 μg/mL representing no PTA treatment) of PTA for 24 h, followed by treatment with 2 μM of DOX for another 48 h. Cell viability was measured using the WST-1 assay. (**B**) PTA did not inhibit DOX-induced cytotoxicity in the human breast cancer cell line MDA-MB-231. MDA-MB-231 cells were pretreated with indicated concentrations of PTA (0 μg/mL representing no PTA treatment) for 24 h, followed by treatment with 2 μM of DOX for 48 h. Cell viability was measured using a WST-1 assay. Data are presented as mean ± standard deviation (SD) of three independent experiments. ### *p* < 0.001 (control vs. DOX) (Student *t*-test); ** *p* < 0.01, and *** *p* < 0.001 (DOX vs. PTA) (Student *t*-test). (**C**) PTA mitigates DOX-induced apoptosis in H9c2 cells. Pretreatment with varying concentrations of PTA (0 μg/mL representing no PTA treatment) for 24 h suppresses DOX-induced (2 µM of DOX for 48 h) expression of apoptosis-related markers, including cleaved-caspase-3 and Bcl-xL, in H9c2 cells. Protein levels were assessed using western blotting with antibodies against cleaved-caspase-3 and Bcl-xL (**left** panel). Band intensities were quantified using Fusion analysis software (Ver. 16.07). The quantified expression was normalized with respect to that of the internal control, β-actin (**middle** and **right** panels). The values are presented as mean ± SD of three independent experiments. The means with different superscript letters are significantly different. ### *p* < 0.001 (control vs. DOX) (Student’s *t*-test); ** *p* < 0.01 (DOX vs. CBW) (one-way ANOVA).

To explore the safeguarding potential of PTA against DOX-triggered cardiotoxicity, H9c2 cells were pre-treated at varying concentrations of PTA (200, 400, 800, and 1600 µg/mL) before exposure to DOX. Assessment using the WST-1 assay revealed that DOX led to a reduction in cell viability by approximately 30%. Notably, PTA pre-treatment at concentrations of 400–1600 µg/mL significantly mitigated DOX-induced decline in H9C2 cell viability in a dose-dependent manner, resulting in cell viability ranging from 50% to 90% (Figure 1A). Furthermore, we investigated whether PTA impacted the anticancer effects of DOX. DOX reduced the viability of the MDA-MB-231 human breast cancer cell line by approximately 40%, a reduction unaffected by PTA treatment. PTA treatment did not exhibit any synergistic effect on DOX-induced cytotoxicity in MDA-MB-231 cells (Figure 1B). To evaluate the effect of PTA on DOX-induced apoptotic cell death, we examined the expression of cleaved-caspase-3 (a pro-apoptotic marker) and Bcl-XL (a mitochondria-dependent anti-apoptotic marker). PTA treatment effectively suppressed DOX-induced caspase-3 cleavage but did not restore Bcl-XL levels in H9c2 cells (*p* < 0.05) (Figure 1C), implying that PTA specifically impedes DOX-induced mitochondria-independent caspase-3 cleavage. In summary, these findings suggest that PTA selectively alleviates DOX-induced cytotoxicity in cardiomyocytes without compromising the anticancer efficacy of DOX.

### 2.2. PTA Protects Cardiac Functions by Inhibiting DOX-Induced Cardiomyocytes Death In Vivo

**Figure 2:**
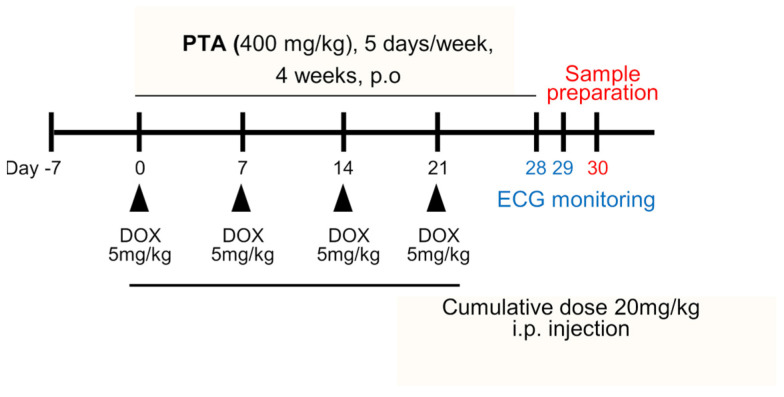
A schematic illustration of the establishment of a mouse model doxorubicin (DOX)-induced cardiotoxicity (DICT). Male C57BL/6 mice received four separated doses of 5 mg/kg DOX via intraperitoneal (i.p) injection at day (D) 0, D7, D14, and D21, over three weeks, resulting in a cumulative dose of 20 mg/kg of DOX. Additionally, 400 mg/kg of PTA or berberine (Ber, positive control) was orally administrated (p.o) to the mice for four weeks. An electrocardiogram was taken 1 week after DOX withdrawal, using a non-invasive method, followed by the collection of blood and cardiac tissue samples a day later.

**Figure 3:**
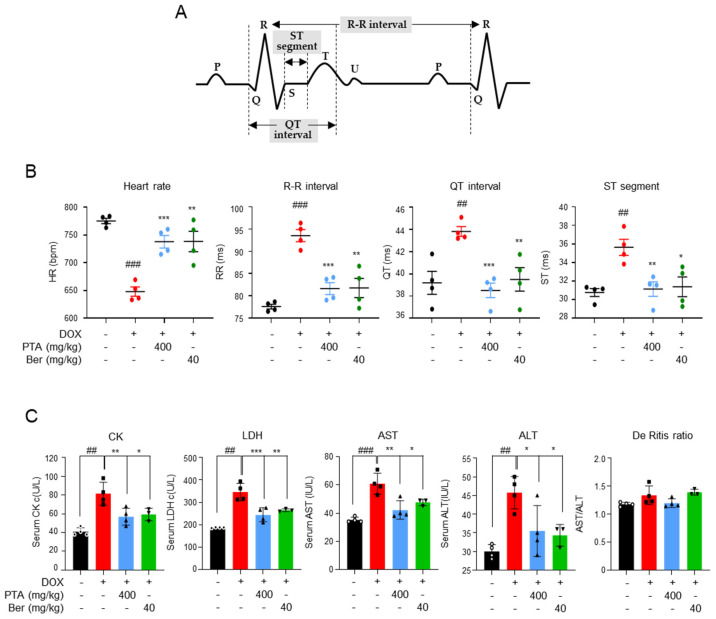
*Poncirus trifoliata* aqueous extract (PTA) protects cardiac function and ameliorates cardiotoxicity in vivo. (**A**) The clinical features of the electrocardiogram. (**B**) PTA ameliorated DICT-induced cardiac dysfunction. ECG was monitored without anesthesia using a non-invasive method. Heat rate, R-R interval, QT interval, and ST segment were measured (*n* = 4/group). (**C**) PTA improved DOX-induced toxicity indicators in the blood. Mice were sacrificed after 10 d of DOX withdrawal, followed by blood collection. Serum levels of creatinine kinase (CK), lactate dehydrogenase (LDH), aspartate aminotransferase (AST), alanine aminotransferase (ALT), and De Ritis ratio (AST/ALT) were measured. Data are presented as mean ± standard error (SE). ## *p* < 0.01, and ### *p* < 0.001 (Student’s *t*-test); * *p* < 0.05, and ** *p* < 0.01, and *** *p* < 0.001 (DOX vs. PTA or Berberine [Ber]) (Student’s *t*-test).

To confirm the in vivo effects of PTA, we established a mouse model of DICT and examined whether PTA can effectively ameliorate DICT-induced cardiac function impairment (Figure 2). DICT was induced in C57BL/6 male mice (8 weeks old) via intraperitoneal injection of 5 mg/kg of DOX over 3 weeks in four separate doses, achieving a cumulative dose of 20 mg/kg of DOX. Mice were orally administered 400 mg/kg of PTA or 40 mg/kg of berberine (Ber, positive control) for 4 weeks [27]. Electrocardiography was performed a week after DOX withdrawal to assess cardiac function parameters, such as heart rate, R-R interval, QT interval, and ST segment (Figure 3A). DOX administration decreased the heart rate of mice in the DOX group (650 beats/min [bpm]) by 120 bpm compared with that in the control group (770 bpm; Figure 3B). However, PTA treatment (400 mg/kg) increased the heart rate of mice with DICT to 740 bpm, which was similar to those of mice in the control and positive control (DOX + Ber, 740 bpm) groups. Additionally, the R-R interval, QT interval, and ST segment were significantly higher in the DOX group than in the control group. Notably, the PTA treatment significantly attenuated DOX-induced increase in the parameters to levels similar to those observed in the control and Ber groups. Furthermore, we investigated the effects of PTA on DOX-induced changes in blood toxicity markers (Figure 3C). Expectedly, DOX administration increased the serum levels of CK and LDH, which are blood markers indicative of cardiac toxicity; however, PTA treatment mitigated this increase. Similar trends were observed in the serum levels of the liver toxicity markers AST and ALT; however, the De Ritis ratio (AST/ALT), a liver injury marker, did not show significant changes in all groups (*p* < 0.05). Collectively, these results indicate that PTA protects against cardiac and hepatic damage and ameliorates DICT-induced impairment in cardiac function. 

### 2.3. PTA Blocks DOX-Induced Cardiac Fibrosis and Death

**Figure 4:**
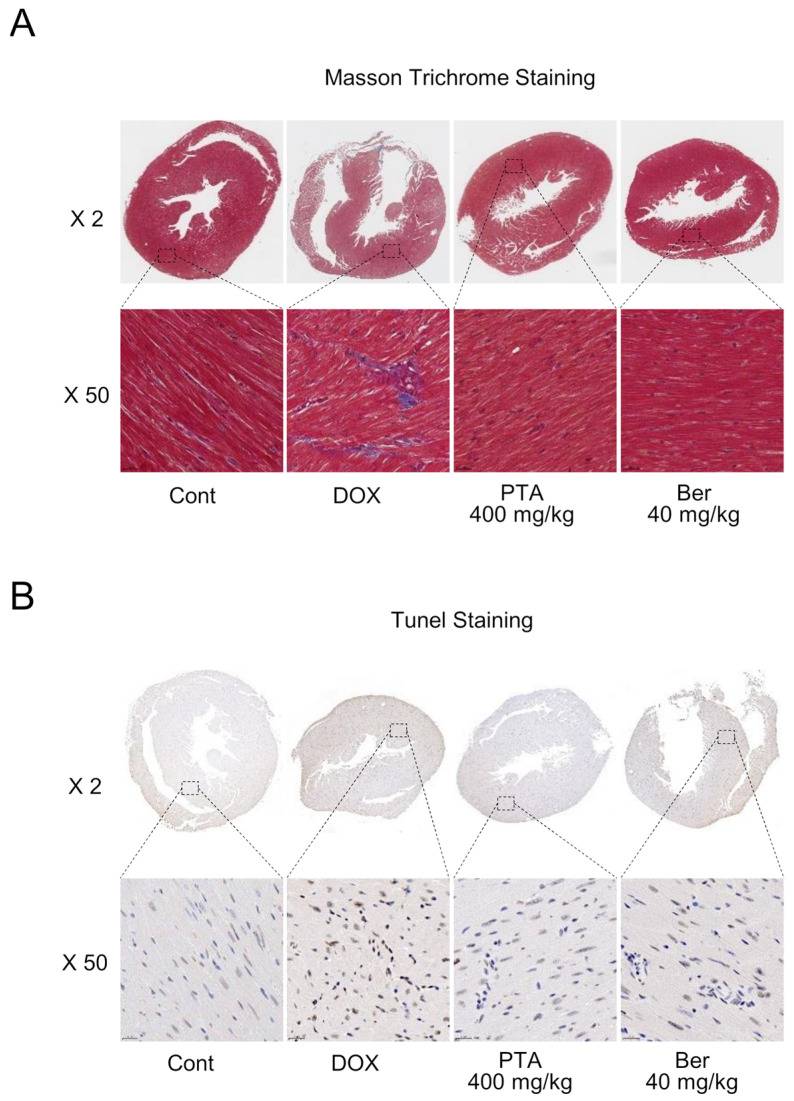
*Poncirus trifoliata* aqueous extract (PTA) alleviates cardiac fibrosis and cardiomyocyte apoptosis in vivo. (**A**) PTA administration reduced doxorubicin (DOX)-induced collagen accumulation in cardiomyocytes. Mice were sacrificed after 10 d of DOX withdrawal, and cardiac tissue samples were collected. Masson’s trichrome staining (MTS) was performed to observe collagen accumulation. The accumulated collagen was stained in blue. (**B**) PTA administration decreased DOX-induced cardiomyocyte apoptosis. Terminal deoxynucleotidyl transferase d UTP nick end labeling (TUNEL) staining was performed to detect apoptotic cells in cardiomyocytes. The apoptotic cells were stained in brown.

Further analysis showed that the protective effects of PTA against DOX-induced cardiac damage were attributable to the inhibition of collagen accumulation within myocardial cells, leading to the prevention of apoptotic cell death. MTS assay showed that DOX administration elevated collagen accumulation in myocardial cells in vivo, which was effectively suppressed and reversed by the PTA treatment (Figure 4A). Moreover, the TUNEL assay indicated that PTA treatment significantly suppressed DICT-induced apoptotic cell death in cardiomyocytes (Figure 4B). Collectively, these results suggest that PTA inhibits the progression of cardiac fibrosis by reducing DICT-induced collagen accumulation within cardiomyocytes, leading to decreased cardiomyocyte apoptosis.

### 2.4. PTA Inhibits DOX-Induced ROS Generation through the Upregulation of Nqo1 Expression

**Figure 5:**
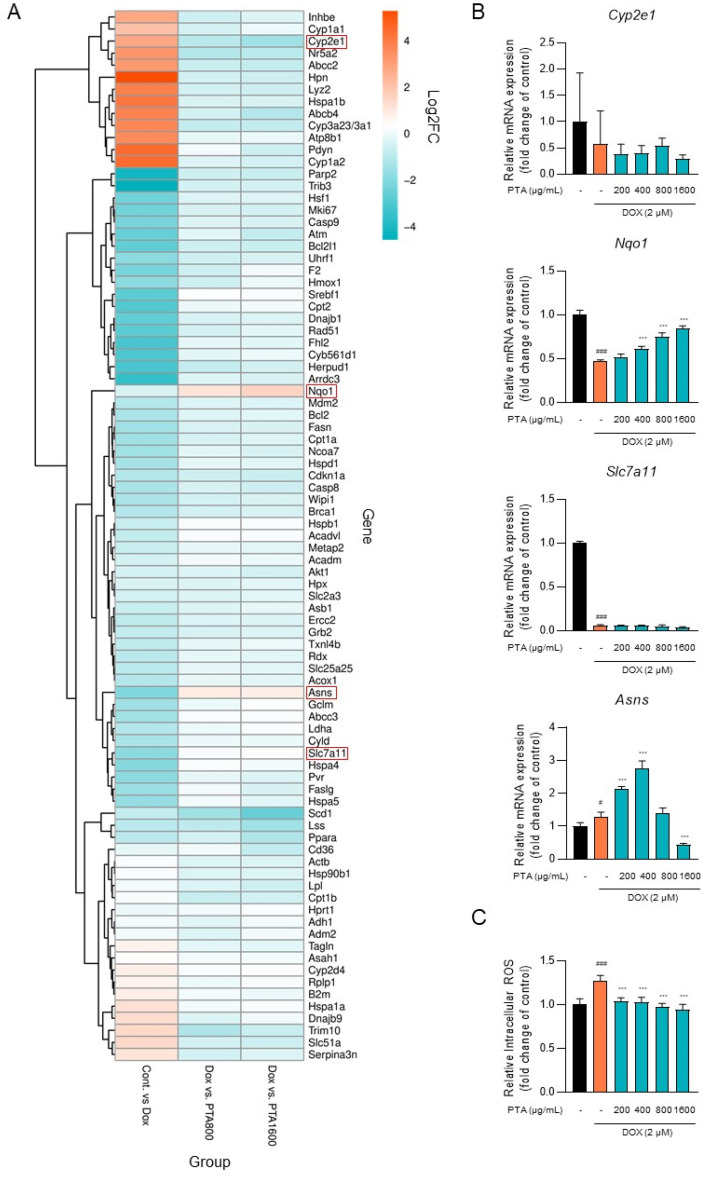
*Poncirus trifoliata* aqueous extract (PTA) inhibits doxorubicin (DOX)-induced reactive oxygen species (ROS) generation by increasing *Nqo1* expression in vitro. (**A**) A PCR microarray was performed on four groups, including control (Cont.), Dox (DOX-treated), PTA800 (800 μg/mL PTA with DOX-treated), and PTA1600 (1600 μg/mL PTA with DOX-treated), using 89 genes involved in cytotoxicity. Heatmaps depicting the fold changes in the expression of the genes in the Cont. vs. Dox, Dox vs. PTA800, and Dox vs. PTA1600 groups were generated using the ‘heatmaply’ function in the R package (ver. 1.4.2) The fold change values were represented as log_2_(fold change) (log_2_FC). (**B**) PTA reverses DOX-induced decreases in *Nqo1* expression in H9c2 cells. The mRNA expression of *Cyp2e1*, *Nqo1*, *Slc7a11,* and *Asns* was quantified using qRT-PCR. The bar graph illustrates the relative mRNA expression of each gene. The values are presented as mean ± SD of three independent experiments. # *p* < 0.05 and ### *p* < 0.001 (control vs. DOX) (Student’s *t*-test); *** *p* < 0.001 (DOX vs. PTA) (one-way ANOVA). (**C**) PTA suppresses DOX-induced intracellular ROS generation in H9c2 cells. Intracellular ROS levels were assessed using CM-H_2_DCFDA reagent. The bar graph illustrates the relative intracellular ROS levels. The values are presented as mean ± SD of three independent experiments. ### *p* < 0.001 (control vs. DOX) (Student’s *t*-test); *** *p* < 0.001 (DOX vs. PTA) (one-way ANOVA).

To investigate the potential therapeutic mechanisms of PTA in DICT, we used a PCR microarray to identify the toxicological pathways affected by PTA. Specifically, we examined the mRNA expression of 89 genes and found that PTA treatment significantly reversed DOX-induced upregulation or downregulation of some genes compared with that in the control group (Figure 5A). Among these genes, *Cyp2e1*, *Nqo1*, *Slc7a11, and Asns* are involved in DICT-related hypertrophy [28], anti-oxidation [29], and ferroptosis [30]. Therefore, we validated the mRNA levels of *Cyp2e1*, *Nqo1*, *Slc7a11*, and *Asns* in H9c2 cells using qRT-PCR. Among the genes, *Nqo1* expression, which was decreased by DOX administration, was restored by the PTA treatment. In contrast, while *Slc7a11* expression was also decreased by DOX treatment, it did not show restoration with PTA treatment. *Cyp2e1* expression was not significantly altered by DOX treatment. *Asns* expression was slightly upregulated by DOX treatment. The PTA treatment further upregulated *Asns* expression until reaching 400 µg/mL and downregulated it at 1600 µg/mL compared to the DOX-treated group without the PTA treatment (Figure 5B). The NQO1 enzyme, encoded by the *Nqo1* gene, is an antioxidant enzyme, and its induction has been associated with a decreased susceptibility to oxidative stress [10]. Moreover, the PTA treatment significantly suppressed DOX-induced intracellular ROS generation in H9c2 cells (Figure 5C). Overall, these results suggest that PTA ameliorates DICT by inhibiting ROS generation through the upregulation of *Nqo1* expression.

### 2.5. Identification of the Chemical Composition of PTA

**Figure 6:**
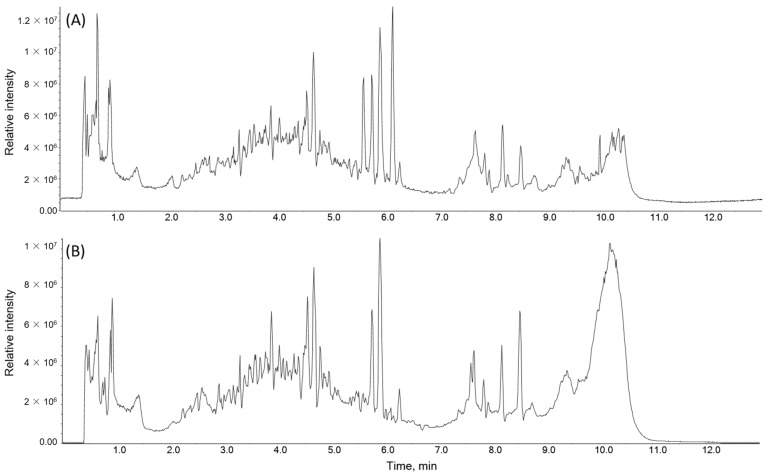
Total ion chromatograms in the (**A**) ESI-positive and (**B**) ESI-negative modes from the UPLC-TQ/MS analysis of *Poncirus trifoliata* aqueous extract (PTA).

A UPLC-TQ/MS analysis was performed to identify the chemical constituents of PTA. A total of 43 compounds, comprising 16 flavonoids, two benzoic acids, three cinnamic acids, three quinic acids, two phenolic glycosides, five amino acids, two organic acids, one sugar, and nine other compounds, were tentatively identified. Among them, naringin, naringenin, choline, naringenin glucoside, and coumaric acid exhibited high relative intensity values exceeding 100,000 in both positive and negative ion modes. In contrast, poncirin, a major compound typically found in immature fruits of *P. trifoliata*, also known as Ponciri Fructus Immaturus, was detected at a lower amount with a relative intensity of 1300 (Table 1 and Figure 6). Naringenin and its glycosides, naringin, and naringenin glucosides, are the major compounds in *P. trifoliata* extracts, [31,32] justifying the high abundance of naringenin and its glucosides in PTA. 

## 3. Discussion

DOX is an effective agent for cancers; however, its clinical application is limited by the high sensitivity of cardiomyocytes to ROS, which are generated following DOX administration [33]. Several mechanisms have been proposed to explain the pathogenesis of DICT, including lipid peroxidation in cardiomyocytes, intracellular calcium dysregulation, immune system dysfunction, and oxidative stress [34]. Although some food materials and phytochemicals possess antioxidant activities, studies have yet to examine their therapeutic potential in DICT. 

Poncirin, a flavonoid glycoside derivative of *P. trifoliata*, attenuates cardiac ischemic injury and enhances H9c2 cell survival following anoxia/reperfusion damage via its antioxidant effects [35]. In the present study, we investigated the therapeutic effects and molecular mechanisms of *P. trifoliata* crude extract in DICT. To the best of our knowledge, this study is the first to demonstrate the potential of *P. trifoliata* and its extract for DICT treatment. Recent studies have reported the inhibitory effects of food ingredients on DICT and their underlying molecular mechanisms. For example, berberine attenuates DOX-induced cardiotoxicity by activating the SIRT1/p66Shc-mediated pathway [27]. Salsolinol ameliorates DOX-induced heart failure by suppressing the mitochondrial calcium uniporter signaling pathway [36]. Fisetin inhibits DOX-induced ferroptosis in cardiomyocytes by upregulating the SIRT1/Nrf2 signaling pathway [37]. Overall, these studies used H9c2 cells as an in vitro model for DICT and demonstrated that each phytochemical increased the survival rate of cells following DOX treatment. The Western blot results suggest that PTA inhibits DOX-induced caspase-3 cleavage without restoring Bcl-XL levels, indicating a specific hindrance of PTA on DOX-induced mitochondria-independent cell death in H9c2 cells. However, the effects of polyphenols on the anticancer activity of DOX have not been examined. Therefore, we examined the effects of PTA on DOX-induced apoptosis in cancer cells in the present study. Neoadjuvant polychemotherapy regimens containing anthracyclines have shown significant effectiveness against triple-negative breast cancer (TNBC), the most malignant subtype of breast cancer [38]. Therefore, we investigated whether PTA interferes with DOX-induced apoptosis in the human breast cancer cell line MDA-MB-231. PTA treatment did not affect the anticancer efficacy of DOX in MDA-MB-231 cells. 

Critical determinants of the phenotypes of murine DICT models include total cumulative dose and duration of administration. These models can be broadly categorized into two types: acute and chronic models. In acute cardiotoxicity, mice or rats receive a one-time dose ranging from 15 to 30 mg/kg [39,40,41,42,43], whereas the chronic cardiotoxicity model involves administering 4–8 fractional doses of 2–5 mg/kg of DOX for 2–4 weeks, amounting to a total cumulative dose of 15–40 mg/kg [44,45,46]. In the present study, DOX administration (5 mg/kg) four times for 4 weeks induced chronic cardiotoxicity in mice. Moreover, the mouse model of DICT demonstrated changes in cardiac electrophysiological features and biomarkers [47,48]. Electrocardiography captures electrical cardiac signals to assess various physiological conditions. Notably, the electrical activity measured from the electrocardiogram is closely related to cardiac function, with a single heartbeat corresponding to a normal sinus cycle on the electrocardiogram, characterized by five distinctive points: P, Q, R, S, and T [49]. The R-R interval (length of the ventricular cardiac cycle), QT interval (time between ventricular depolarization and repolarization), and ST segment (plateau phase of ventricular repolarization) can provide valuable insights into cardiac diseases [50,51]. DOX administration increased the R-R interval, QT interval, and ST segment in the mice, indicating a deterioration in cardiac function. Additionally, serum biomarkers, such as serum CK, LDH, AST, and ALT, were significantly upregulated in the in vivo DICT model. Functional abnormalities in the heart and elevated serum toxicity indicators result from DICT-induced myocardial fibrosis and apoptosis [52]. Notably, our examination of the De Ritis ratio revealed no significant changes following DOX administration in the in vivo model, indicating that DOX treatment may result in non-specific or early-stage liver damage [53]. Masson’s trichrome and TUNEL assays were performed to investigate myocardial cell fibrosis and apoptosis, respectively. DOX administration increased myocardial cell fibrosis and apoptosis in the experimental mice. Overall, these results confirm the successful establishment of the DICT model [54,55]. 

In this investigation, the in vivo efficacy of PTA was evaluated using a mouse model of Doxorubicin-induced cardiotoxicity (DICT). DICT was induced by intraperitoneal injection of DOX, and mice were subsequently orally administered PTA or berberine (used as a positive control) for four weeks. Berberine served as a positive control due to its established efficacy in mitigating doxorubicin-induced cardiomyocyte toxicity through various mechanisms in previous studies [27,56,57]. Electrocardiography conducted a week after DOX withdrawal revealed significant cardiac dysfunction, characterized by a reduced heart rate, and altered ECG parameters. Notably, we recently reported that the water extract of *Capsella bursa-pastoris* mitigates doxorubicin-induced myocardial injury, as evidenced by ECG parameters [58]. Remarkably, the PTA treatment at 400 mg/kg effectively restored the heart rate and ameliorated DOX-induced alterations in R-R interval, QT interval, and ST segment, aligning with the control and positive control groups. Analysis of blood toxicity markers demonstrated that DOX elevated cardiac toxicity markers (CK and LDH), which were attenuated by the PTA treatment. Furthermore, PTA mitigated DOX-induced increases in liver toxicity markers (AST and ALT), highlighting its protective effects against both cardiac and hepatic damage. These findings collectively underscore the protective role of PTA in mitigating DICT-induced impairment in cardiac function, suggesting its potential as a therapeutic agent against doxorubicin-associated cardiotoxicity.

In our exploration of the potential therapeutic mechanisms of PTA in DICT, we employed PCR microarray analysis to scrutinize the toxicological pathways influenced by the DOX and PTA treatment. Among the 89 genes analyzed, 24 genes exhibited alterations in expression due to the DOX treatment, and these tendencies were notably restored by PTA treatment. Our focus centered on key genes implicated in DOX-induced cardiotoxicity, including *Cyp2e1*, *Nqo1*, and *Slc7a11* [28,29,30], along with the evaluation of the Asns gene, associated with ferroptosis Remarkably, *Nqo1* expression, reduced by DOX administration, was effectively restored by the PTA treatment. Additionally, the PTA treatment significantly suppressed DOX-induced intracellular ROS generation in H9c2 cells, highlighting its antioxidative properties. The protective effects of PTA against DICT can be attributed to its capacity to mitigate oxidative stress, a recognized major contributor to the onset of doxorubicin-induced cardiotoxicity, as supported by several studies [7,59]. DOX, a quinone compound, is converted to a semiquinone structure that provides unstable electrons to oxygen in the mitochondrial electron transport chain complex I and consequently generates superoxide anions (O_2_^−^) [60]. O_2_^−^ can be detoxicated into relatively stable low-toxicity hydrogen peroxide (H_2_O_2_) by antioxidant enzymes such as superoxide dismutases (SODs) [61], and H_2_O_2_ can be further oxidized to H_2_O by catalase and glutathione peroxidases (GPXs) [62]. H_2_O_2_ subsequently undergoes a reaction with ions through the Fenton reaction, generating a highly reactive hydroxyl radical (·OH) [34]. Therefore, DICT can be alleviated by increasing the activity of antioxidant enzymes to inhibit ROS generation. Notably, various natural extracts and phytochemicals can upregulate the expression and activities of antioxidant enzymes, leading to a decrease in ROS production and normalization of mitochondrial functions, which can inhibit DOX-induced cardiomyocyte apoptosis [63]. Although the antioxidant effects of *P. trifoliata* have been established in the literature [64,65], this is the first study to report that the antioxidant effects of PTA contribute to the alleviation of DICT. Particularly, the PTA treatment attenuated DOX-induced NQO1 inhibition. NQO1, a phase II antioxidant enzyme, converts the quinone structure of DOX into a stable hydroquinone [66], and a decrease in NQO1 is an early biomarker of DICT [9]. Nrf2 is an important antioxidant factor with a vital role in counteracting oxidative stress and is also involved in suppressing DICT [67,68,69]. Accumulated Nrf2 in the cytoplasm translocates to the nucleus, where it targets genes, including NQO1 and HO-1, which contain the antioxidant response element region [70]. Glycyrrhetinic acid and genistatin ameliorate oxidative damage and cardiomyocyte apoptosis by activating the Nrf2-HO-1 signaling pathway [71,72]. Baicalein mitigates DICT-induced cardiomyocyte damage by upregulating Nrf2 and NQO1 expression [73]. Additionally, indole-3 carbinol accelerates Nrf2 activity, inducing NQO1 expression in the cardiac tissue of mice with DICT [74]. Conclusively, the upregulation of the NQO1 axis may represent a possible strategy for mitigating DICT. 

The chemical composition of PTA was thoroughly scrutinized through UPLC-TQ/MS analysis, revealing the presence of 43 tentatively identified compounds. This diverse array encompassed 16 flavonoids, two benzoic acids, three cinnamic acids, three quinic acids, two phenolic glycosides, five amino acids, two organic acids, one sugar, and nine other compounds. Particularly noteworthy were naringin, naringenin, choline, naringenin glucoside, and coumaric acid, showcasing high relative intensity values in both positive and negative ion modes. The prevalence of naringenin and its glycosides in PTA aligns with prior research on *P. trifoliata* extracts [31,32]. Poncirin, a compound prominent in immature fruits of *P. trifoliata*, was detected at a lower concentration with low relative intensity, consistent with previous studies noting its decline as the fruit matures [75]. *Ponciri Fructus*, derived from the dried immature fruits of *P. trifoliata*, is recognized to contain poncirin at a high concentration [69]. The identified compounds present a diverse chemical profile, implying potential multifaceted biological activities of PTA, with the lower relative intensity of poncirin underscoring the impact of the maturity stage of the raw material on chemical composition. These findings substantially contribute to a comprehensive understanding of PTA’s chemical constituents, laying the foundation for further investigations into its therapeutic potential and pharmacological applications.

Although this study suggests the potential for controlling DICT through the antioxidant activity of *P. trifoliata* and highlights NQO1 as a key factor, it has some limitations. For example, although the mouse model of DICT was well established, it did not totally recapitulate the electrophysiological characteristics of the human heart. Therefore, studies involving human induced pluripotent stem cell-derived CMs (hiPSC-CM) are necessary [76]. Moreover, it is imperative to validate the protective effects of PTA using hiPSC-CMs, which closely mimic the characteristics of human cardiomyocytes. Additionally, although we were able to identify the constituents of PTA using UPLC-TQ/MS, we did not examine the roles of the 42 identified compounds in ameliorating DICT. We also acknowledge the impact of the low number of animals in our in vivo experiments on the reliability of statistical conclusions, and we appreciate your feedback in emphasizing the need for more robust statistical evaluations in future investigations. Comprehensive research addressing these limitations would facilitate the adoption of PTA for the treatment of life-threatening side effects of anthracycline-based chemotherapeutic agents, especially cardiotoxicity.

## 4. Materials and Methods

### 4.1. Plant Materials, Preparation, and Extraction

The *P. trifoliata* was acquired from an agricultural product brand in the Republic of Korea. Briefly, 2 kg of mature fruit components were cleaned, subjected to a 2 h hot water extraction at 95 °C under vacuum conditions, and then centrifuged at 8000× g for 30 min. The resulting extract was freeze-dried using a vacuum-tray freeze dryer and preserved at −20 °C until needed. 

### 4.2. Cell Culture and Chemicals

The H9c2 (rat myoblasts) and MDA-MB-231 (human breast carcinoma) cells were sourced from the American Type Culture Collection (Manassas, VA, USA). H9c2 cells were sustained in Dulbecco’s Modified Eagle Medium (DMEM; Gibco-BRL, MD, USA), while MDA-MB-231 cells were cultured in Roswell Park Memorial Institute (RPMI) 1640 medium (Gibco-BRL), supplemented with 10% fetal bovine serum (FBS; Gibco-BRL) and 1% antibiotic–antimycotic (Gibco-BRL), within a humidified chamber at 37 °C under 5% CO_2_ conditions. Doxorubicin was procured from ApexBio (A1832, Houston, TX, USA), and berberine chloride was obtained from Sigma-Aldrich (B3251, St. Louis, MO, USA).

### 4.3. Cell Viability Assay

The H9c2 or MDA-MB-231 cells (1 × 10^4^ cells/well) were plated in 96-well plates and allowed to incubate for 24 h. To explore the protective effects of PTA against DOX-induced cellular toxicity, the cells were pre-exposed to PTA for 24 h, followed by treatment with 2 µM DOX for 48 h. Subsequently, the cells were treated with a 10 µL solution of water-soluble tetrazolium salt (WST-1) from Enzo Life Sciences, Inc. (Farmingdale, NY, USA) for 2 h. Absorbance was then measured within the 450–650 nm range using a microplate reader from Molecular Devices (Sunnyvale, CA, USA).

### 4.4. Immunoblot Analysis 

Cell extracts were gathered using M-PER Lysis buffer (Thermo Fisher Scientific), which included phosphatase and protease inhibitors from Roche (Basel, Switzerland), and incubated for 5 min at room temperature. Subsequently, the lysates were subjected to centrifugation at 20,000× g for 20 min at 4 °C and utilized for immunoblot assays. The antibodies employed in this investigation were anti-cleaved caspase-3 (Asp175; Cat. No. 9664S; Cell Signaling Technology), Bcl-xL (Cat. No. 2764S; Cell Signaling Technology), and β-actin (Cat. No. 4967S; Cell Signaling Technology). The target proteins’ expression was visualized using enhanced chemiluminescence (ECL, Thermo Scientific, Rockford, IL, USA) with an imaging system (Vilber Lourmat, ZAC de Lamirault, France). Protein expression levels were normalized to β-actin (used as an internal control), and band quantification was performed using Fusion analysis software Ver. 16.07 (Vilber Lourmat).

### 4.5. Quantitative Real-Time Polymerase Chain Reaction (PCR)

Total RNA was isolated using RNAiso Plus (Takara, Kusatsu, Shiga, Japan) and subjected to reverse transcription through a cDNA synthesis kit (Toyobo, Osaka, Japan), according to the provided manufacturer’s instructions. Subsequently, gene amplification was carried out via a CFX Connect Real-Time PCR Detection System (Bio-Rad, Hercules, CA, USA) with SYBR Green PCR Master Mix (Roche, Mannheim, Germany) and specific primers. The primers (5′ to 3′) utilized in this study included rat *Cyp2e1* (forward, ATGAGTTTTCTGGACGGGGG; reverse, GGAAAACCTCCG-CACATCCT), *Nqo1* (forward, GGATGGGAGGTGGTCGAATC; reverse, GCTCCCCTGTGATGTCGTTT), *Slc7a11* (forward, ACCCAAGTGGTTCAGACGAT; reverse, GGGCAGATGGCCAAGGATTT), *Asns* (forward, GCACAAGACCAGCCGTAATTG; reverse, GCGCAATCTTCATCGCACTC), and *Actb* (forward, TCCACCCGCGAG-TACAACC; reverse, GACGACGAGCGCAGCGATA). The 2^ΔΔCT^ method was used to calculate the relative expression levels of the target genes, normalized to Actb (utilized as an internal control).

### 4.6. Microarray Analysis of Gene Expression

A PCR microarray was performed utilizing the Rat Molecular Toxicology PathwayFinder RT^2^ Profiler PCR array kit (Qiagen, Valencia, CA, USA), according to the manufacturer’s instructions provided. In summary, cDNA derived from total RNA was introduced into the individual wells of a 96-well plate pre-equipped with primers for 89 genes. Gene amplification was carried out using the CFX Connect Real-Time PCR Detection System (Bio-Rad, Hercules, CA, USA) along with SYBR Green PCR Master Mix (Roche, Mannheim, Germany).

### 4.7. Measurement of Intracellular ROS Generation

The H9c2 cells were plated in 96-well plates at a density of 1 × 10^4^ cells/well, and then pre-treated with 200, 400, 800, or 1600 μg/mL of PTA a day later. After 24 h, the cells were exposed to 2 μM of DOX for an additional 24 h. For the assessment of intracellular ROS levels, cells were treated with 2 μM of CM-H_2_DCFDA reagent (Invitrogen™) for 30 min, and the resulting fluorescence intensity at 485/530 nm (excitation/emission) was gauged using a microplate reader (Molecular Devices, Sunnyvale, CA, USA).

### 4.8. In Vivo Cardiotoxicity Model

All the animal experiments were adherent to the International Animal Care Protocols and were approved by the Korea Food Research Institute (IACUC Approval No. KFRI-M-22004). Eight-week-old C57BL/6 mice were acclimatized for one week before the experiment and randomly divided into four groups (*n* = 3 or 4/group). The mice were kept in a controlled environment (humidity: 40–60%; temperature: 23 ± 2 °C) following a 12 h light/dark cycle. To induce cardiotoxicity, mice received intraperitoneal injections of 5 mg/kg DOX for 4 weeks, resulting in a cumulative dose of 20 mg/kg. PTA (400 mg/kg) and berberine (40 mg/kg) were orally administered five days a week, with berberine serving as the positive control. Electrocardiograms (ECG) were analyzed using EzCG Analysis Software (BIOPAC Systems Inc., Goleta, CA, USA) two days before sacrifice. Blood and cardiac tissues were collected from the mice to evaluate DOX-induced cardiac injury.

### 4.9. Blood Test

Blood serum was isolated by centrifuging at 3000× g for 10 min at 4 °C. The concentrations of creatine kinase (CK), lactate dehydrogenase (LDH), aspartate aminotransferase (AST), and alanine aminotransferase (ALT) in the serum were measured using a blood chemistry analyzer (DRI-CHEM 3500s, Fujifilm, Tokyo, Japan), according to the manufacturer’s protocol.

### 4.10. Masson’s Trichrome Staining and Terminal Deoxynucleotidyl Transferase dUTP Nick-End Labeling (TUNEL) Assay

Cardiac specimens from mice were fixed in 4% buffered formalin, embedded in paraffin, and sectioned into 4–5-μm-thick slices. Masson’s trichrome staining was carried out using an MT kit (StatLab, American Master Tech, Lodi, CA, USA) following the manufacturer’s instructions provided. Following deparaffinization and rehydration, the sections underwent immersion in Bouin’s fluid at 4 °C for 1 h, staining with Weigert’s hematoxylin, incubation in Biebrich scarlet acid fuchsin phosphomolybdic/phosphotungstic acid and aniline blue, and subsequent fixation with 1% acetic acid. The stained slides were then scanned using a Panoramic Digital Slide Scanner (Gaia Science, E Pasir, Singapore), and images were captured using a Slide Converter (3DHISTECH Ltd., Budapest, Hungary).

### 4.11. Ultra-Performance Liquid Chromatography (UPLC)-Triple Quadrupole Mass Spectrometry (TQ/MS) (UPLC-TQ/MS) Analysis 

The analysis of PTA using UPLC-TQ/MS was conducted on an Agilent 1290 Infinity system (Agilent Technologies, Palo Alto, CA, USA) coupled with a SCIEX 4500 TQ/MS instrument (Sciex, Framingham, MA, USA). Chromatographic separation was achieved using an Acquity UPLC HSS T3 column (2.1 mm × 5 mm, 1.7 μm; Waters, Milford, MA, USA) maintained at 40 °C. An injection volume of 5 µL was used. The mobile phase, composed of eluents A (0.1% formic acid in water) and B (0.1% formic acid in acetonitrile), flowed at a rate of 0.45 mL/min. The gradient elution proceeded as follows: 0–1 min: 1–5% B; 1–3 min: 5–25% B; 3–4.8 min: 25–35% B; 4.8–5.8 min held at 35% B; 5.8–6.8 min: 35–45% B; 6.8–7.8 min held at 45% B; 7.8–8.8 min: 45–60% B; 8.8–9.3 min: 60–100% B; 9.3–10 min: 100–1% B, followed by a 3 min hold to re-equilibrate the column. The autosampler and column oven temperatures were set at 4 and 40 °C, respectively. 

### 4.12. Statistical Analysis

The data are presented as mean ± standard deviation (SD). To assess significant differences, a one-way analysis of variance (ANOVA) was employed, followed by Dunnett’s post-hoc test. Statistical significance was set at * *p* < 0.05, ** *p* < 0.01, *** *p* < 0.001, and **** *p* < 0.0001. All the statistical analyses were carried out using GraphPad Prism 9 software (Ver. 9.0.2).

## Figures and Tables

**Table 1:** Metabolites tentatively identified by the UPLC-QTOF-MS in *Poncirus trifoliate*.

No	Compound	Class	Rt (min)	Ionization Mode	Molecular Formula	Observed Precursor Ions (*m/z*)	Difference (ppm) ^a^	Relative Intensity
1	Quercetin dihexose	Flavonoids	3.46	[M + H]+	C_27_H_30_O_17_	627.1566	0.13	42,440
2	Kaempferol dihexose		3.64	[M + H]+	C_27_H_30_O_16_	611.1623	0.15	56,436
3	Isorhamnetin sophorose or dihexose		3.69	[M + H]+	C_28_H_32_O_17_	641.1728	0.14	56,243
4	Quercetin pentose glucuronide		4.26	[M − H]−	C_26_H_26_O_17_	609.1462	0.48	77,546
5	Quercetin hexose		4.36	[M − H]−	C_21_H_20_O_12_	463.0889	−0.14	14,069
6	Kaempferol rutinoside		4.52	[M − H]−	C_27_H_30_O_15_	593.1529	−0.09	35,315
7	Poncirin		4.53	[M − H]−	C_28_H_33_O_14_	593.1843	4.55	1300
8	Naringin		4.68	[M − H]−	C_27_H_32_O_14_	579.1719	−0.13	701,538
9	Naringenin		4.68	[M + H]+	C_15_H_12_O_5_	273.0756	0.26	261,691
10	Naringenin glucoside		4.83	[M − H]−	C_21_H_22_O_10_	433.1144	−0.16	110,209
11	Eriodictyol		5.17	[M − H]−	C_15_H_12_O_6_	287.0562	−0.25	18,712
12	Catechin		6.71	[M − H]−	C_15_H_14_O_6_	289.0715	−0.26	3088
13	Kaempferol malonylhexose		6.80	[M + H]+	C_24_H_22_O_14_	535.1451	0.82	3087
14	Quercetin		6.92	[M − H]−	C_15_H_10_O_7_	301.0716	0.96	2067
15	Kaemprerol		6.95	[M + H]+	C_15_H_10_O_6_	287.0548	0.25	3304
16	Isorhamnetin		7.22	[M − H]−	C_16_H_12_O_7_	315.0515	−0.22	5558
17	Vanillic acid	Benzoic acid	3.02	[M − H]−	C_8_H_8_O_4_	167.0346	−0.46	7575
18	Protocatechuic acid		3.08	[M − H]−	C_7_H_6_O_4_	153.0190	−0.50	4152
19	Cinnamic acid	Cinnamic acid	2.77	[M − H]−	C_9_H_8_O_2_	147.0448	−0.52	132
20	Coumaric acid		4.32	[M − H]−	C_9_H_8_O_3_	163.0395	−0.48	107,500
21	Ferulic acid		4.55	[M − H]−	C_10_H_10_O_4_	193.0502	−0.40	8523
22	Quinic acid	Quinic acid	0.66	[M + H]+	C_7_H_12_O_6_	193.0706	−4.97	76,818
23	Coumaroyl quinic acid		3.48	[M − H]−	C_16_H_18_O_8_	337.0929	−0.22	168,809
24	Feruloyl quinic acid		3.66	[M − H]−	C_17_H_20_O_9_	367.1038	−0.19	17,189
25	Caffeic acid hexose	Phenolic glycosides	3.30	[M − H]−	C_15_H_18_O_9_	341.0883	−0.20	2500
26	Coumaric acid hexose		3.61	[M − H]−	C_15_H_18_O_8_	325.0937	−0.20	47,655
27	Asparagine	Amino acid	0.53	[M + H]+	C_4_H_8_N_2_O_3_	133.0603	0.52	45,090
28	Valine		0.87	[M + H]+	C_5_H_11_NO_2_	118.0859	0.58	85,271
29	Leucine		2.03	[M + H]+	C_6_H_13_NO_2_	132.1014	0.51	9718
30	Tyrosine		2.04	[M + H]+	C_9_H_11_NO_3_	182.0812	0.40	21,774
31	Phenylalanine		2.77	[M − H]−	C_9_H_11_NO_2_	164.0715	−0.46	3986
32	Malic acid	Organic acid	0.78	[M − H]−	C_4_H_6_O_5_	133.0137	−0.59	93,348
33	Succinic acid		1.74	[M − H]−	C_4_H_6_O_4_	117.0189	−0.65	1182
34	Glucose	Sugar	0.59	[M − H]−	C_6_H_12_O_6_	179.0560	−0.41	19,976
35	Choline	Other	0.54	[M]+	C_5_H_14_NO	104.1062	−0.13	160,124
36	Shikimic acid		0.86	[M − H]−	C_7_H_10_O_5_	173.0454	−0.43	5159
37	Ascorbic acid		1.35	[M + H]+	C_6_H_8_O_6_	175.0171	−0.85	120
38	3-Hydroxy-3-methylglutaric acid		2.25	[M − H]−	C_6_H_10_O_5_	161.0456	−0.45	3463
39	Chlorogenic acid		3.20	[M − H]−	C_16_H_18_O_9_	353.0882	−0.19	13,847
40	Tryptophan		3.27	[M − H]−	C_11_H_12_N_2_O_2_	203.0824	−0.37	3919
41	Kynurenic acid		3.43	[M + H]+	C_10_H_7_NO_3_	190.0497	0.37	13,904
42	Caffeoyl shikimic acid		3.93	[M − H]−	C_16_H_16_O_8_	335.0783	−0.19	4748
43	Scopoletin		4.59	[M − H]−	C_10_H_8_O_4_	191.0347	−0.40	45,206

^a^ Values in the ‘Difference (ppm)’ column denote the measurement error, with lower ppm indicating higher accuracy. Higher ppm values reflect greater measurement deviation.

## Data Availability

Data is available in the manuscript.
